# Extracorporeal Carbon Dioxide Removal Using a Renal Replacement Therapy Platform to Enhance Lung-Protective Ventilation in Hypercapnic Patients With Coronavirus Disease 2019-Associated Acute Respiratory Distress Syndrome

**DOI:** 10.3389/fmed.2020.598379

**Published:** 2020-11-12

**Authors:** Faeq Husain-Syed, Horst-Walter Birk, Jochen Wilhelm, Claudio Ronco, V. Marco Ranieri, Bianka Karle, Stefan Kuhnert, Khodr Tello, Matthias Hecker, Rory E. Morty, Susanne Herold, Oliver Kehl, Hans-Dieter Walmrath, Werner Seeger, István Vadász

**Affiliations:** ^1^Divison of Nephrology, Department of Internal Medicine II, University Hospital Giessen and Marburg, Justus Liebig University Giessen, Giessen, Germany; ^2^Division of Pulmonary and Critical Care Medicine, Department of Internal Medicine II, University Hospital Giessen and Marburg, Justus Liebig University Giessen, Giessen, Germany; ^3^International Renal Research Institute of Vicenza, San Bortolo Hospital, Vicenza, Italy; ^4^Universities of Giessen and Marburg Lung Center, Justus Liebig University Giessen, Giessen, Germany; ^5^The Cardio-Pulmonary Institute, Giessen, Germany; ^6^Institute for Lung Health, Justus Liebig University Giessen, Giessen, Germany; ^7^Department of Medicine (DIMED), Università di Padova, Padua, Italy; ^8^Department of Medical and Surgical Sciences (DIMEC), Anaesthesia and Intensive Care Medicine, Sant'Orsola-Malpighi Hospital, Alma Mater Studiorum University of Bologna, Bologna, Italy; ^9^Department of Lung Development and Remodelling, Max Planck Institute for Heart and Lung Research, Bad Nauheim, Germany

**Keywords:** continuous renal replacement therapy, respiratory acidosis, SARS-CoV-2, extracorporeal organ support, respiratory dialysis

## Abstract

Coronavirus disease 2019 (COVID-19)-associated acute respiratory distress syndrome (ARDS) is associated with high mortality. Lung-protective ventilation is the current standard of care in patients with ARDS, but it might lead to hypercapnia, which is independently associated with worse outcomes. Extracorporeal carbon dioxide removal (ECCO_2_R) has been proposed as an adjuvant therapy to avoid progression of clinical severity and limit further ventilator-induced lung injury, but its use in COVID-19 has not been described yet. Acute kidney injury requiring renal replacement therapy (RRT) is common among critically ill COVID-19 patients. In centers with available dialysis, low-flow ECCO_2_R (<500 mL/min) using RRT platforms could be carried out by dialysis specialists and might be an option to efficiently allocate resources during the COVID-19 pandemic for patients with hypercapnia as the main indication. Here, we report the feasibility, safety, and efficacy of ECCO_2_R using an RRT platform to provide either standalone ECCO_2_R or ECCO_2_R combined with RRT in four hypercapnic patients with moderate ARDS. A randomized clinical trial is required to assess the overall benefit and harm.

**Clinical Trial Registration:** ClinicalTrials.gov. Unique identifier: NCT04351906.

## Introduction

The percentages of coronavirus disease 2019 (COVID-19) patients diagnosed with acute respiratory distress syndrome (ARDS) range between 20 and 67% of hospitalized patients (1, 2) and 100% of mechanically ventilated patients (3) and are associated with high mortality (2). Lung-protective ventilation is the current standard of care for ARDS (4), which limits ventilator-induced lung injury but may lead to elevated carbon dioxide (CO_2_) levels and respiratory acidosis, which are independently associated with worse outcomes in the setting of ARDS (5, 6). In these patients, extracorporeal CO_2_ removal (ECCO_2_R) may help to avoid the progression of clinical severity (5). Acute kidney injury (AKI) is common among critically ill COVID-19 patients, with ~20% requiring renal replacement therapy (RRT) (7). Recent studies have proposed the integration of ECCO_2_R into continuous RRT (CRRT) platforms to provide combined CO_2_ removal and renal support using low blood-flow levels (<500 mL/min) (5, 8). Of note, only one study described the use of CRRT platform-driven ECCO_2_R without hemofilter to provide standalone ECCO_2_R in patients with mild to moderate ARDS. However, that trial used an ECCO_2_R membrane with a significantly lower surface area (0.32 m^2^ as opposed to 1.35 m^2^ in the current study), limiting the rate of maximal CO_2_ removal (9). In centers with available dialysis, low-flow ECCO_2_R using CRRT platforms might be an option to efficiently allocate resources for patients with hypercapnia as the main indication. The use of ECCO_2_R has not been described so far in COVID-19-associated ARDS.

## Materials and Methods

We report results of a single-center study evaluating the feasibility and safety of ECCO_2_R in combination with a CRRT platform as a standalone therapy or combined with CRRT for ARDS patients with refractory hypercapnia (arterial partial pressure of CO_2_ [PaCO_2_] > 55 mmHg) secondary to confirmed COVID-19 to effectively decrease CO_2_ levels and enhance lung-protective ventilation.

### Study Design and Participants

COVID-19 was diagnosed according to the World Health Organization (WHO) guidance (10). All patients were nursed in an isolation intensive care unit (ICU) with other patients suffering from COVID-19. The study was prospectively registered at http://clinicaltrials.gov (Identifier: NCT04351906). Patients were sedated with fentanyl, midazolam, and propofol. Other medications, including antibiotics, fluids, catecholamines, and transfusions, were left to the discretion of the attending physician.

### Participants

In-patients ≥18 years of age with confirmed COVID-19 admitted to the University Hospital Giessen and Marburg, Giessen Medical Center, were enrolled in the feasibility study. Inclusion criteria were mild-to-moderate ARDS according to the Berlin definition (11), 100 mmHg < partial alveolar oxygen pressure/fraction of inspired oxygen (PaO_2_/FiO_2_) <300 mmHg with positive end-expiratory pressure >5 cmH_2_O on mechanical ventilation expected to last >24 h; hypercapnia >55 mmHg with or without metabolic acidosis (pH < 7.3); bilateral opacities on chest imaging; with or without AKI requiring dialysis. Exclusion criteria were age <18 years, pregnancy, patients with decompensated heart failure or acute coronary syndrome, respiratory acidosis with persistent partial pressure of blood carbon dioxide (PaCO_2_) levels >80 mmHg, acute brain injury, severe liver insufficiency (Child–Pugh scores > 7) or fulminant hepatic failure, decision to limit therapeutic interventions, catheter access to a femoral vein or jugular vein impossible, and pneumothorax.

### Extracorporeal Carbon Dioxide Removal Operational Characteristic

ECCO_2_R was provided using a polymethylpentene, hollow fiber, gas-exchanger membrane (multiECCO_2_R; Eurosets, Medolla, Italy), a labeled and certified European device to be used in conjunction with multiFiltrate CRRT platforms (Fresenius Medical Care, Bad Homburg, Germany) for combined respiratory and renal support. The manufacturer determined the multiECCO_2_R membrane's maximum duration to be 72 h. A 13.5-Fr dual lumen hemodialysis catheter (Niagara, Bard Access, Heidelberg, Germany) was percutaneously inserted under in the femoral vein. Sweep gas flow was set at a gas/blood flow ratio of 15:1. Data were collected before starting ECCO_2_R (baseline) and 1, 4, 24, and 48 h after initiation of ECCO_2_R. A bloodline warmer (Barkey S-line) and a thermal pad (both from Barkey, Leopoldshöhe, Germany) wrapped around the multiECCO_2_R, as well as a warming blanket, were used to avoid undercooling of the patient.

**Figure 1:**
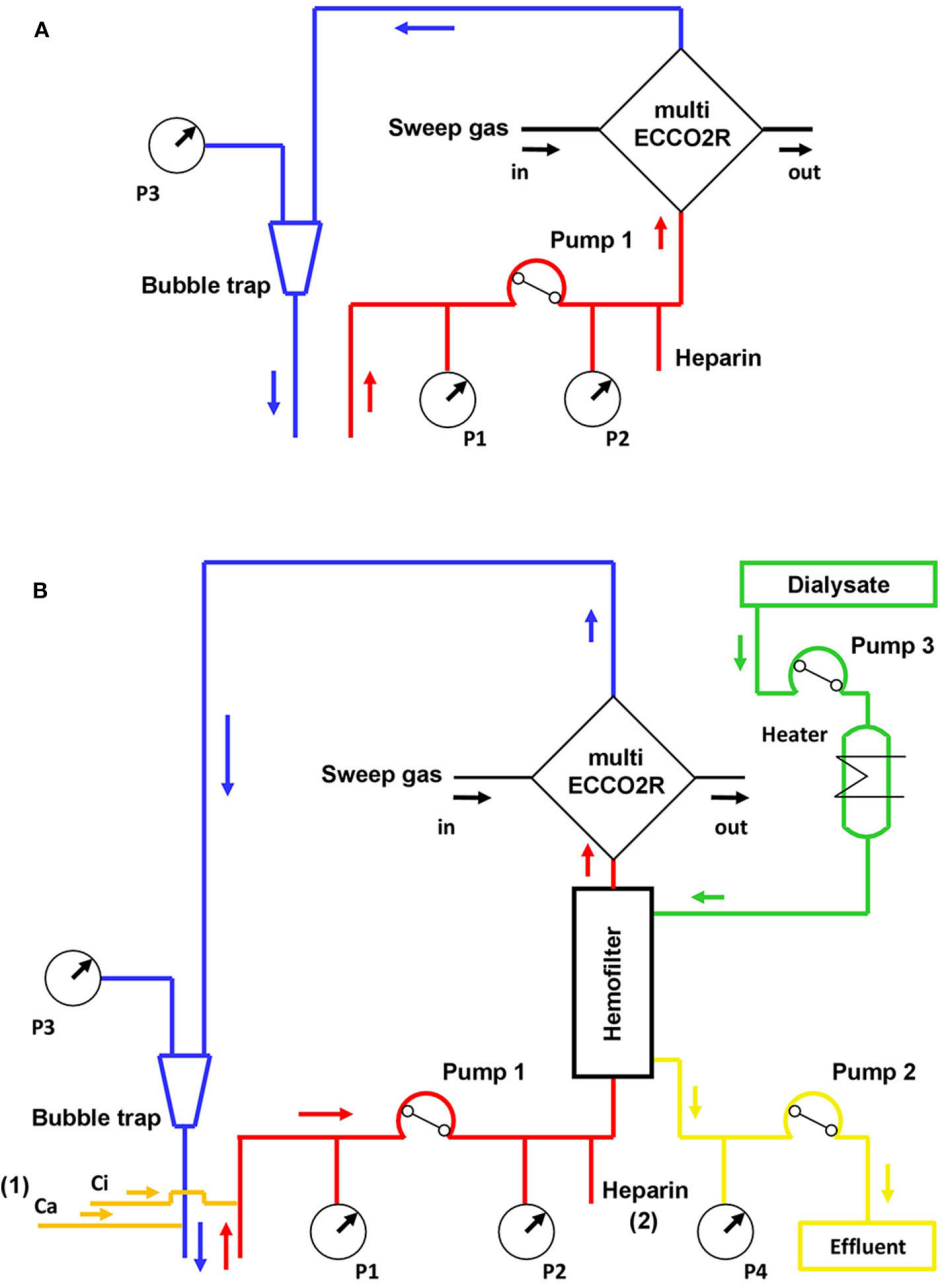
Schematic representation of the ECCO_2_R circuit either as standalone therapy **(A)** or in-line after the hemofilter for combined ECCO_2_R and RRT **(B)**. ECCO_2_R in conjunction with RRT may be performed with regional citrate anticoagulation (1) or systemic heparinization (2). Ca, calcium chloride solution; Ci, trisodium citrate solution; Pump 1, blood line; Pump 2, effluent dialysate line; Pump 3, dialysate line; P1, in-flow pressure sensor; P2, pre-filter pressure sensor; P3, out-flow pressure sensor; P4, effluent dialysate pressure sensor; RRT, renal replacement therapy.

Figure 1 depicts a schematic representation of the ECCO_2_R setup used in this study, either as standalone therapy (Figure 1A) or in conjunction with RRT (Figure 1B). The technical terminology of the extracorporeal circuit was based on a nomenclature developed for RRT (12). For standalone ECCO_2_R, the multiFiltrate was set in hemoperfusion mode. ECCO_2_R was commenced at a blood flow of 400 mL/min. Systemic heparinization was started after catheter insertion aiming for an activated partial thromboplastin time of 60–80 s.

For ECCO_2_R + CRRT, the multiFiltrate was set in continuous venovenous hemodialysis (CVVHD) mode, and the multiECCO_2_R was inserted in series after the hemofilter (Ultraflux AV 1000S, Fresenius Medical Care, Bad Homburg, Germany). ECCO_2_R + CRRT was commenced at a blood flow of 200 mL/min. CVVHD was delivered with an effluent dose of 25 mL/kg/h and regional citrate anticoagulation aiming a post-filter ionized calcium concentration of ~0.25–0.35 mMol/L.

### Definitions

Lung-protective ventilation strategies were the standard of care for invasive mechanical ventilation (4). Treatment strategies for COVID-19-associated ARDS were based on the WHO interim guidance (10), which were in line with our institutional standard of care for other forms of ARDS. Of note, at the time of patient recruitment, the WHO guidance on corticosteroids to treat patients with severe and critical COVID-19 was not available (13). Therefore, we did not routinely use corticosteroids for this patient population. Severe adverse events were defined as recently described (14). The feasibility of ECCO_2_R was assessed using Bowen et al.'s (15) feasibility framework. The use of RRT was at the discretion of the attending physician rather than by predefined biochemical or clinical criteria. However, RRT was initiated emergently when life-threatening changes in fluid, electrolyte, and acid-base balance occurred (16). The Institute of Medical Virology (Justus Liebig University Giessen, Germany) processed nasopharyngeal swabs and bronchoalveolar lavage fluid specimens, and severe acute respiratory syndrome coronavirus 2 infection was confirmed by real-time PCR according to the previously described protocols (17).

## Results

We report data of four male patients (median age: 62 [range, 52–74] years) admitted to our ICU between April and May 2020 due to ARDS secondary to confirmed severe acute respiratory syndrome coronavirus 2 infection (for clinical data, see Table 1). After implementing adjunctive measures for ARDS, all patients showed an improvement in oxygenation (PaO_2_/FiO_2_ ratio); however, in the later course of intensive care, all patients developed severe hypercapnia despite escalated ventilation parameters. In patients 1 and 3, hypercapnia was seen as the result of diffuse consolidations and fibrotic remodeling of the lungs as indicated by the low compliance (18.4 mL/mbar), whereas patients 2 and 4 developed hypercapnia, at least in part, secondary to underlying chronic obstructive pulmonary disease.

**Table 1:** Characteristics of four patients with COVID-19 before ECCO_2_R initiation.

	**Patient 1**	**Patient 2**	**Patient 3**	Patient 4^*^
**Demographics**				
Sex	Male	Male	Male	Male
Age, years	57	74	67	52
Body mass index, kg/m^2^	29.4	24.3	26.8	42.1
**Comorbidities**	Hypertension, diabetes	Hypertension, diabetes, CAD, COPD, CKD	Hypertension, diabetes, CAD, CKD	Hypertension, diabetes, COPD, CKD
**Clinical characteristics**				
SAPS II	37	51	54	43
SOFA score	7	9	8	11
ICU length of stay before ECCO_2_R initiation, days	23	6	25	8
Pre-ECCO_2_R adjuvant therapy				
Prone positioning	Yes	Yes	Yes	Yes
Nitric oxide	Yes	No	Yes	Yes
Duration of ECCO_2_R, days	6	4	5	8
V_T_, mL/kg PBW	5.6	7.2	6.5	7.3
RR, breaths/min	30	19	31	21
V_E_, L/min	10.3	10.1	10.5	13.8
P_PLAT_, cmH_2_O	30	26	31	27
PEEP, cmH_2_O	10	11	6	11
Driving pressure, cmH_2_O	20	15	25	16
Compliance, mL/mbar	18.4	34.6	18.4	41.2
PaO_2_/FiO_2_ ratio	153.3	150.6	160.0	140.0
PaCO_2_, mmHg	57.4	70.0	56.6	58.7
pH	7.38	7.29	7.41	7.23
Arterial ${\text{HCO}}_{3^{-}}$, mMol/L	33.3	32.3	35.1	21.4
LVEF, %	60	40	65	60
Norepinephrine dose, μg/kg/min	0.002	0.336	0.219	0.038
**Laboratory findings**				
White cell count, g/L	7.1	12.9	26.3	17.1
Total lymphocytes	1.54	0.94	1.94	1.26
Hemoglobin, g/dL	84	95	90	93
Platelet count, giga/L	316	357	288	301
Creatinine, mg/dL^†^	0.5	1.9	1.0	2.0
Urea, mg/dL^‡^	37	197	101	230
Lactate dehydrogenase, U/L	311	492	237	365
Alanine aminotransferase, U/L	104	378	40	212
Aspartate aminotransferase, U/L	44	359	35	363
Albumin, g/L	24.9	23.6	29.2	29.7
B-type natriuretic peptide, pg/mL	48	591	93	9
C-reactive protein, mg/L	71.1	164.5	113.9	197.7
Procalcitonin, μg/L	0.5	6.1	7.5	1.6
Interleukin-6, μg/L	74	2150	95	55
Ferritin, μg/L	1588	2107	723	1076
D-dimer, mg/L	3.1	15.3	1.77	3.9

* *Patient received ECCO_2_R + CRRT*.

† *To convert the values for serum creatinine to mg/dL, multiply by 88.4*.

‡ *To convert the value for urea to blood urea nitrogen, multiply by 0.467*.

*CAD, coronary artery disease; CKD, chronic kidney disease; COPD, chronic obstructive pulmonary disease; COVID-19, coronavirus disease 2019; CRRT, continuous renal replacement therapy; ECCO_2_R, extracorporeal carbon dioxide removal; FiO_2_, fraction of inspired oxygen; ${\text{HCO}}_{3}^{-}$, bicarbonate; ICU, intensive care unit; LVEF, left ventricular ejection fraction; NA, not applicable/not available; PaCO_2_, arterial partial pressure of carbon dioxide; PaO_2_, arterial partial pressure of oxygen; PBW, predicted body weight; PEEP, positive end-expiratory pressure; P_PLAT_, plateau pressure; RR, respiratory rate; SAP, Simplified Acute Physiology Score; SOFA, Sequential Organ Failure Assessment; V_E_, minute volume; V_T_, tidal volume*.

**Figure 2:**
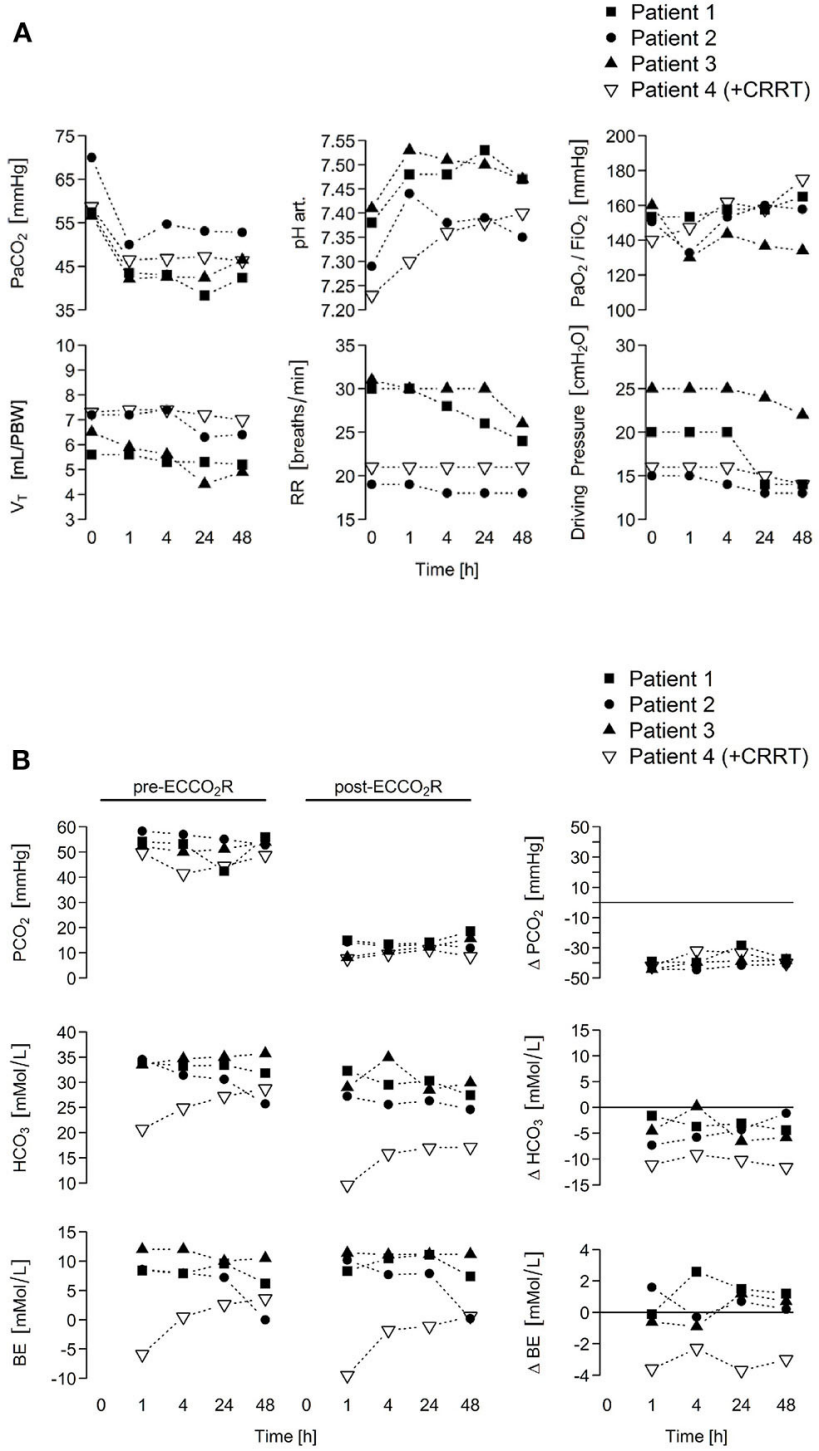
ECCO_2_R rapidly normalizes arterial hypercapnia in patients with ARDS secondary to COVID-19, allowing de-escalation of ventilatory parameters. **(A)** To enhance carbon dioxide removal, ECCO_2_R was applied with a constant blood flow of 400 mL/min (patients 1–3) or 200 mL/min (patient 4; combined with CRRT) administering a sweep gas flow at a gas/blood flow ratio of 15:1 (6 or 3.5 L/min, respectively). Time course of blood gases and ventilator parameters is depicted. **(B)** Pre- to post-ECCO_2_R changes in PCO_2_, bicarbonate, and base excess levels in all four patients that simultaneously points as in **(A)** are shown upon ECCO_2_R therapy. ARDS, acute respiratory distress syndrome; BE, base excess; COVID-19, coronavirus disease 2019; CRRT, continuous renal replacement therapy; ECCO_2_R, extracorporeal carbon dioxide removal; FiO_2_, fraction of inspired oxygen; HCO_3_, bicarbonate; PaCO_2_, arterial partial pressure of carbon dioxide; PBW, predicted body weight; PCO_2_, venous partial pressure of carbon dioxide; PaO_2_, arterial partial pressure of oxygen; RR, respiratory rate; V_E_, minute volume; V_T_, tidal volume.

ECCO_2_R was implemented at a blood-flow rate of 400 mL/min in patients 1–3, resulting in a PaCO_2_ decrease from a median 57.4 [56.6–70.0] to 43.5 [42.1–50.0] mmHg within 1 h, whereas pH increased from a median 7.38 [7.29–7.41] to 7.48 [7.44–7.53] mmHg within 1 h (Table 2). Patient 4 developed combined respiratory and metabolic acidosis secondary to hypercapnia and AKI, and ECCO_2_R + CRRT was commenced with a blood-flow rate of 200 mL/min, leading to a decrease of PaCO_2_ from 58.7 to 46.5 mmHg within 1 h while pH and bicarbonate levels progressively increased. CRRT ultrafiltration (100 mL/h) was started at 38 h post-ECCO_2_R initiation due to oliguria. Tidal volume, plateau and driving pressure, as well as respiratory rate could be reduced during the second day of ECCO_2_R (from median 6.9 [5.6–7.3] to 5.8 [4.9–7.0] mL/kg PBW, median 28.5 [26.0–31.0] to 24.5 [22.0–28.0] cmH_2_O, median 18.0 [15.0–25.0] to 14.0 [13.0–22.0] cmH_2_O, and median 25.5 [19.0–31.0] to 21.5 [18.0–26.0] breaths/min, respectively; Figure 2A). The PaO_2_/FiO_2_ ratio remained unchanged throughout the study period (from median 152.0 [140.0–160.0] to 161.4 [134.0–175.1]). There was no detectable impact of ECCO_2_R on hemodynamics and vasopressor support. A comparison of pre- and post-ECCO_2_R PCO_2_ values showed a ~30 mmHg decrease (Figure 2B). No patient- or ECCO_2_R/CRRT-related adverse events occurred. Downtime ranged from 2 to 8% of the total treatment time owing due to the turning of patients into the prone position. In all four patients, the ECCO_2_R treatment could be terminated after a median of 5.5 (4.5–7.5) days due to a sustained improvement in hypercapnia. In patient 4, however, CRRT was continued for another 4 days due to oliguria. Furthermore, patient 2 developed AKI stage 3, necessitating CRRT 6 days after the termination of ECCO_2_R as a sequel to septic shock.

**Table 2:** Individual time course of operational characteristics, blood gas, ventilatory, and hemodynamic parameters during ECCO_2_R.

	**Patient 1**					**Patient 2**					**Patient 3**					Patient 4^*^				
**Operational characteristics**	**Baseline**	**1 h**	**4 h**	**24 h**	**48 h**	**Baseline**	**1 h**	**4 h**	**24 h**	**48 h**	**Baseline**	**1 h**	**4 h**	**24 h**	**48 h**	**Baseline**	**1 h**	**4 h**	**24 h**	**48 h**
Blood flow, mL/min	400	400	400	400	400	400	400	400	400	400	400	400	400	400	400	200	200	200	200	200
Sweep gas flow, L/min	6	6	6	6	6	6	6	6	6	6	6	6	6	6	6	3.5	3.5	3.5	3.5	3.5
CRRT ultrafiltration rate, mL/h	NA	NA	NA	NA	NA	NA	NA	NA	NA	NA	NA	NA	NA	NA	NA	0	0	0	0	100^†^
CRRT effluent rate, mL/kg/h	NA	NA	NA	NA	NA	NA	NA	NA	NA	NA	NA	NA	NA	NA	NA	25	25	25	25	25
aPTT, s	30	NA	67	98	93	48	80	79	79	NA	85	NA	NA	56	65	27	26	NA	26	25^‡^
**Blood gas parameters**																				
**Arterial**																				
PaCO_2_, mmHg	57.4	43.5	43.0	38.3	42.4	70.0	50.0	54.7	53.1	52.8	56.6	42.1	42.6	42.4	46.5	58.7	46.5	46.8	47.2	46.3
pH	7.38	7.48	7.48	7.53	7.47	7.29	7.44	7.38	7.39	7.35	7.41	7.53	7.51	7.50	7.47	7.23	7.30	7.36	7.38	7.40
PaO_2_, mmHg	69.2	63.0	71.0	71.0	66.0	68.0	66.0	69.0	74.0	62.0	80.0	65.0	79.0	82.0	79.0	77.0	81.0	89.0	71.0	94.0
${\text{HCO}}_{3^{-}}$, mMol/L	33.3	32.0	31.6	31.9	30.8	32.3	33.1	32.3	32.4	28.1	35.1	34.6	33.8	33.0	33.3	21.4	21.9	25.9	27.2	27.8
BE, mMol/L	7.6	8.3	7.9	8.7	6.9	4.8	8.2	6.2	6.6	2.5	9.4	10.8	10.2	9.1	8.9	−5.5	−3.9	0.8	2.3	3.0
**Pre-ECCO**_**2**_**R**																				
PCO_2_, mmHg	NA	54.2	53.2	42.5	55.9	NA	58.3	57.0	55.1	52.8	NA	52.4	50.0	51.2	54.0	NA	49.8	41.4	44.5	48.7
${\text{HCO}}_{3^{-}}$, mMol/L	NA	33.9	33.2	33.4	31.8	NA	34.5	31.4	30.6	25.7	NA	33.5	34.7	35.0	35.7	NA	20.7	24.9	27.2	28.7
BE, mMol/L	NA	8.4	7.9	9.6	6.2	NA	8.6	8.0	7.2	0.0	NA	12.0	12.0	10.0	10.5	NA	−5.9	0.5	2.6	3.6
**Post-ECCO**_**2**_**R**																				
PCO_2_, mmHg	NA	15.0	13.4	14.1	18.6	NA	14.3	12.5	13.6	11.9	NA	8.2	10.7	12.4	15.7	NA	7.6	9.5	11.3	8.5
${\text{HCO}}_{3^{-}}$, mMol/L	NA	32.3	29.5	30.3	27.4	NA	27.2	25.6	26.3	24.6	NA	29.0	34.9	28.5	29.9	NA	9.6	15.8	17.0	17.1
BE, mMol/L	NA	8.3	10.5	11.1	7.4	NA	10.2	7.7	7.9	0.2	NA	11.4	11.1	11.2	11.2	NA	−9.5	−1.8	−1.1	0.6
**Ventilator parameters**																				
V_T_, mL/kg PBW	5.6	5.6	5.3	5.3	5.2	7.2	7.2	7.4	6.3	6.4	6.5	5.9	5.6	4.4	4.9	7.3	7.4	7.4	7.2	7.0
RR, breaths/min	30	30	28	26	24	19	19	18	18	18	31	30	30	30	26	21	21	21	21	21
V_E_, L/min	10.3	10.6	10.8	10.7	7.5	10.1	9.5	9.2	8.6	9.4	10.5	11.5	10.0	9.2	6.2	13.8	13.8	13.9	13.7	13.4
P_PLAT_, cmH_2_O	30	30	29	22	22	26	26	25	24	24	31	31	31	30	28	27	27	27	26	25
PEEP, cmH_2_O	10	10	9	8	8	11	11	11	11	11	6	6	6	6	6	11	11	11	11	11
Driving pressure, cmH_2_O	20	20	20	14	14	15	15	14	13	13	25	25	25	24	22	16	16	16	15	14
Compliance, mL/mbar	18.4	18.5	17.5	24.9	24.5	34.6	32.5	27.1	33.2	31.4	18.4	15.8	14.3	13.7	13.6	41.2	41.4	41.5	43.5	45.6
PaO_2_/FiO_2_ ratio	153.3	153.3	157.8	157.8	165.0	150.6	132.7	153.3	160.0	157.8	160.0	130.0	143.6	136.7	134.0	140.0	147.3	161.8	157.8	175.1
**Hemodynamic parameters**																				
Mean arterial pressure, mmHg	71	74	74	76	75	64	89	82	69	66	79	79	77	74	66	63	62	66	67	77
Heart rate, beats/min	93	83	84	92	78	70	58	66	72	70	105	95	92	90	88	84	83	76	72	86
Norepinephrine dose, μg/kg/min	0.002	0.002	0.002	0.002	0.002	0.336	0.336	0.420	0.428	0.430	0.219	0.274	0.192	0.205	0.207	0.038	0.038	0.038	0.038	0.038

* *Patient received ECCO_2_R + CRRT*.

† *CRRT ultrafiltration was started at 38 h post-ECCO_2_R initiation*.

‡ *ECCO_2_R + CRRT was performed with regional citrate anticoagulation*.

*aPTT, activated partial thromboplastin time; BE, base excess; COVID-19, coronavirus disease 2019; CRRT, continuous renal replacement therapy; ECCO_2_R, extracorporeal carbon dioxide removal; FiO_2_, fraction of inspired oxygen; ${\text{HCO}}_{3^{-}}$, bicarbonate; PaCO_2_, arterial partial pressure of carbon dioxide; PCO_2_, venous partial pressure of carbon dioxide; PaO_2_, arterial partial pressure of oxygen; PBW, predicted body weight; PEEP, positive end-expiratory pressure; P_PLAT_, plateau pressure; RCA, regional citrate anticoagulation; RR, respiratory rate; NA, not applicable/not available; V_E_, minute volume; V_T_, tidal volume*.

## Discussion

Our data indicate that low-flow ECCO_2_R using CRRT platforms might be safe and feasible to provide either standalone ECCO_2_R or ECCO_2_R combined with CRRT. This minimally invasive approach leads to efficient CO_2_ removal in the setting of moderate ARDS. No patient- or ECCO2R/CRRT-related adverse events occurred. Importantly, these data also implicate that every ICU with available dialysis may apply RRT platform-driven ECCO_2_R to limit ventilator-induced lung injury or rescue uncontrollable respiratory acidosis even in situations where “standard” ECCO_2_R consoles are not available. To the best of our knowledge, this is the first description of ECCO_2_R in COVID-19. Although these data may provide the rationale for randomized clinical trials, the following limitations need to be acknowledged. Given the invasive nature of an ECCO_2_R therapy, future randomized trials are required to assess the overall benefit and harm before widespread implementation can be recommended. Also, eligibility criteria should be further examined, particularly in those without an indication for CRRT. Furthermore, if the ECCO_2_R is intended to be continuous, sustaining a blood flow of 400 mL/min with a temporary catheter may be challenging, particularly in patients with COVID-19 who are obese or require prone positioning. COVID-19 induces a hypercoagulable state in many patients, which may result in premature extracorporeal circuit failure (18). No studies are available to date to aid in the selection of anticoagulation strategy, in particular when introducing an extracorporeal circulation. Thus, close monitoring of the extracorporeal circuit performance is advisable to ensure maximal circuit patency, as the initial anticoagulation strategy may not be effective in all patients, and a stepwise escalation and/or alternative plans (e.g., combination of different anticoagulation strategies) may be required. However, if using CRRT, we suggest CVVHD or continuous venovenous hemodiafiltration to decrease filtration fraction and reduce the risk of circuit clotting (19). In addition, COVID-19-associated ARDS may follow uncontrolled host immune response to the virus with the release of various immune mediators, especially cytokines, damage-associated molecular patterns, and pathogen-associated molecular patterns (20, 21). Extracorporeal blood purification techniques (e.g., hemoperfusion; RRT with surface-modified AN69, polymethylmethacrylate, or high-cut off membranes) have been proposed as adjuvant therapy for critically ill patients with COVID-19 to restore immune homeostasis through the removal of these circulating mediators (7). As many healthcare agencies have authorized emergency use of various extracorporeal blood purification techniques, these treatments might be indicated as sequential extracorporeal therapies in special cases in which immuno-dysregulation is evident, inflammatory parameters or cytokines are elevated, and other supportive therapies are failing or insufficient. Nonetheless, careful patient selection is required if these are to be used, as the benefits and adverse effects in COVID-19 patients have not been formally studied. Finally, additional costs associated with the use of ECCO_2_R in conjunction with RRT platforms in COVID-19-associated ARDS may be offset by a potential cost reduction through the elimination of daily rental costs for standalone ECCO_2_R consoles, the recruitment of dialysis professionals in centers with available dialysis to operate ECCO_2_R, and a shorter length of ICU and hospital stay. However, large, multicenter randomized clinical trials are required to support the cost–benefit ratio of ECCO_2_R in conjunction with RRT platforms.

In conclusion, our data indicate that low-flow ECCO_2_R using CRRT platforms might be safe and feasible to provide either standalone ECCO_2_R or ECCO_2_R combined with CRRT. A multicenter randomized trial is warranted to assess the effects of CRRT platform-driven ECCO_2_R on clinical outcomes of patients with ARDS secondary to COVID-19 or other pathogenic factors.

## Data Availability Statement

The datasets used and/or analyzed during the study are available from the corresponding author upon reasonable request.

## Ethics Statement

The study protocol was approved by the Ethics Committee of the Medical Faculty of the Justus Liebig University Giessen (AZ 63/20) and complied with the Declaration of Helsinki. Legally authorized representatives of the patients provided written informed consent.

## Author Contributions

FH-S, H-WB, CR, H-DW, WS, and IV: concept and design of the study. VR, BK, SK, KT, MH, RM, SH, and OK: literature research and clinical advice. FH-S, H-WB, JW, CR, VR, BK, SK, KT, MH, RM, SH, OK, H-DW, WS, and IV: acquisition, analyses, or interpretation of data, and critical revision of the manuscript for important intellectual content. FH-S and IV: manuscript drafting and had full access to all study data and had final responsibility for submitting for publication. FH-S, JW, and IV: figure illustration. H-WB, H-DW, WS, and IV: study supervision. All authors shared the study design, data collection, data analyses, data interpretation, as well as preparation, review, and approval of the manuscript.

## Conflict of Interest

WS received personal fees for consulting from Bayer Pharma, Liquidia Technologies, and United Therapeutics outside the submitted work. The remaining authors declare that the research was conducted in the absence of any commercial or financial relationships that could be construed as a potential conflict of interest.
